# Deaths in Immigration and Customs Enforcement (ICE) detention: A Fiscal Year (FY) 2021–2023 update

**DOI:** 10.3934/publichealth.2024011

**Published:** 2024-02-27

**Authors:** Cara Buchanan, Sameer Ahmed, Joseph Nwadiuko, Annette M. Dekker, Amy Zeidan, Eva Bitrán, Thomas Urich, Briah Fischer, Elizabeth R.E. Burner, Parveen Parmar, Sophie Terp

**Affiliations:** 1 Department of Emergency Medicine, Harvard Medical School, Harvard University, Boston, MA, USA; 2 Department of Emergency Medicine, Keck School of Medicine, University of Southern California, Los Angeles, CA, USA; 3 Department of Medicine, UCLA David Geffen School of Medicine, Los Angeles, CA, USA; 4 Department of Health Policy and Management, UCLA Fielding School of Public Health, Los Angeles, CA, USA; 5 Department of Emergency Medicine, UCLA David Geffen School of Medicine, Los Angeles, CA, USA; 6 Department of Emergency Medicine, Emory University School of Medicine, Atlanta, GA, USA; 7 American Civil Liberties Union of Southern California, Los Angeles, CA, USA; 8 Department of Obstetrics and Gynecology, University of Southern California, Los Angeles General Medical Center, Los Angeles, CA, USA

**Keywords:** immigration detention, immigration health, COVID-19, correctional health, public health

## Abstract

**Background:**

This study describes the deaths of individuals in Immigration and Customs Enforcement (ICE) detention between FY2021–2023, updating a report from FY2018–2020, which identified an increased death rate amidst the COVID-19 pandemic.

**Methods:**

Data was extracted from death reports published online by ICE. Causes of deaths were recorded, and death rates per 100,000 admissions were calculated using population statistics reported by ICE. Reports of individuals released from ICE custody just prior to death were also identified and described.

**Results:**

There were 12 deaths reported from FY2021–2023, compared to 38 deaths from FY2018–2020. The death rate per 100,000 admissions in ICE detention was 3.251 in FY2021, 0.939 in FY2022, and 1.457 in FY2023, compared with a pandemic-era high of 10.833 in FY2020. Suicide caused 1 of 12 (8.3%) deaths in FY2021–2023 compared with 9 of 38 (23.7%) deaths in FY2018–2020. COVID-19 was contributory in 3 of 11 (25%) medical deaths in FY2021–2023, compared with 8 of 11 (72.7%) in the COVID-era months of FY2020 (p = 0.030). Overall, 4 of 11 (36.3%) medical deaths in FY2021–2023 resulted from cardiac arrest in detention facilities, compared with 6 of 29 (20.3%) in FY2018–2020. Three deaths of hospitalized individuals released from ICE custody with grave prognoses were identified.

**Conclusions:**

The death rate among individuals in ICE custody decreased in FY2021–2023, which may be explained in part by the release of vulnerable individuals following recent federal legal determinations (e.g., *Fraihat v. ICE*). Identification of medically complex individuals released from ICE custody just prior to death and not reported by ICE indicates that reported deaths underestimate total deaths associated with ICE detention. Attentive monitoring of mortality outcomes following release from ICE custody is warranted.

## 1. Introduction

The 2018 Department of Homeland Security (DHS) Appropriations Bill set a requirement for US Immigration and Customs Enforcement (ICE) to publicly report all in-custody deaths within 30 days, with subsequent reporting to be completed and published within 60 days of the initial report, beginning in Fiscal Year (FY) 2018 [1]. Since then, ICE has released death reports following each in-custody death since April 2018 [2],[3].

Prior work evaluating deaths in ICE detention between FY2018–2020 found an increased death rate amidst the COVID-19 pandemic, during which potentially preventable causes of death including COVID-19 and suicide contributed to more than half of total deaths [4]. Since the prior study, immunizations that effectively prevent mortality from COVID-19 have become widely available [5], and the average daily population of individuals in ICE detention has declined each year [6]. Furthermore, in April 2020, the court case *Fraihat v. ICE* determined that ICE's institutional response to the COVID-19 pandemic was systematically deficient and ICE was ordered to 1) identify and track people with risk factors associated with poor outcomes from COVID-19 infection, 2) consider custody redeterminations for any detained people with these risk factors, and 3) release individuals from custody if not able to protect them based on individual vulnerabilities [7]. Although the ruling has since been overturned on an appeal, many individuals released via *Fraihat v. ICE* and related COVID-19 litigation (*Roman v. Wolf, Zepeda Rivas v. Jennings*) remain out of custody. In light of this changing landscape, this study provides an interim update to describe deaths in ICE detention between FY2021–2023 as compared with those previously described between FY2018–2020 [4].

## 2. Methods

This exploratory case series updates findings from a prior publication [4] describing causes of and circumstances surrounding deaths among individuals in ICE detention using data extracted from publicly available death reports mandated by the 2018 DHS Appropriations Bill [1],[2]. All individuals with a death report issued between FY2021–2023 were included, adding to data from prior FY2018–2020 published reports. Deaths of all individuals in ICE custody are included regardless of the location where the death occurred (e.g., inside a detention facility or during inpatient hospitalization). To ensure completeness of the dataset, the published list of ICE death reports was cross-referenced with a search of news releases issued by independent media and immigrant advocacy organizations regarding deaths of individuals in ICE custody [2],[8]–[12]. Data extracted from death reports include demographic information (age, gender), length of time in ICE detention, identified cause of death, and location of death.

Consistent with prior work, cause of death was categorized as related to suicide or medical causes. All medical deaths were reviewed for instances where COVID-19 was listed as a cause of or contributory to death. Instances where individuals died of a cardiac arrest occurring due to a primary medical condition (e.g., not secondary to suicide) were identified, along with the physical location of the cardiac arrest. Also recorded were the number of days in ICE custody prior to terminal hospital transfer, and whether a detained individual's death was pronounced (1) by Emergency Medical Services (EMS) in the detention center; (2) within 60 minutes of arrival to the emergency department (ED); or (3) during hospital admission. Duration of terminal hospitalization was recorded. For medical deaths that occurred in the detention center, initiation of cardiopulmonary resuscitation (CPR) and the application and use of automated external defibrillator (AED) pads was recorded.

Death rates per person-year as well as per 100,000 admissions were calculated for FY2021–2023 using mathematical methods from prior studies evaluating deaths among detained immigrants in the United States; pertinent formulas are included in the notes section of Table 1 [13]. The total number of deaths and average daily population in ICE detention were obtained from reports posted on the ICE website [2],[14],[15]. As part of the case series analysis, characteristics were compared between medical and suicide deaths, along with those between the two study periods of FY2018–2020 and FY2021–2023. Student's t-tests were used to compare age. Negative binomial regressions were used to compare duration in ICE custody, and among those hospitalized prior to death, the duration of hospitalization from the date of departure from the detention facility. Finally, Fischer's exact tests were used to calculate proportions. Statistical analyses were performed using Stata/MP16.1 (Stata Corp. 2021. College Station, TX). This analysis of publicly available documents pertaining to deceased subjects was determined not to require human subjects' approval by the Institutional Review Board at the University of Southern California. Though names of individuals who died are visible in public documents, the authors of this study elected to redact names and specific identifiers in this manuscript.

Additionally, reports of individuals released from ICE custody just prior to death were also identified and described [8]–[12],[16]. Two illustrative case studies are included as supplementary exhibits (Exhibit 1,2). One instance involved an individual who died in ICE custody for which a public death report was available to review, and another who died shortly after release from ICE custody for which a public death report was not available. Each summarizes events surrounding the death as well as findings of subsequent investigations based on published death reports (when available), and information obtained from media reports, public records, and legal documents.

## 3. Results

A summary of deaths in ICE detention between FY2018–2023 are included in Table 1. Overall, there were 12 deaths in FY2021–2023, compared to 38 deaths during the prior study period. The death rate per 100,000 admissions in ICE custody was 3.251 in FY2021, 0.939 in FY2022, and 1.457 in FY2023, compared to 2.303 in FY2018, 1.499 in FY2019, and 10.833 in FY2020. Overall, the average daily population (ADP) of individuals in ICE custody trended down during the most recent study period, whereas the average length of stay (ALOS) initially down-trended and then approached the pre-pandemic baseline between FY2021–2023.

**Table 1.** Death rates in US Immigration and Customs Enforcement (ICE) Custody FY 2018 to 2023 (n = 50).

**Year**	**Deaths**	**Average Daily Population (ADP), n**	**Average Length of Stay (ALOS), d**	**Admissions per Year, n**	**Death Rate per Person Year**	**Death Rate per 100,000 Admissions**
2018	9	42,188	39.4	390,828	0.021	2.303
2019	8	50,165	34.3	533,826	0.016	1.499
2020	21	33,724	63.5	193,847	0.062	10.833
2021	5	19,254	45.7	153,779	0.026	3.251
2022	3	22,577	25.8	319,403	0.013	0.939
2023	4	28,131	37.4	274,541	0.014	1.457

Note: This table uses methodology used by prior investigators evaluating death rate per 100,000 admissions to ICE detention. Average Daily Population (ADP) and Average Length of Stay (ALOS) in ICE custody were obtained from reports obtained from the ICE website. Consistent with prior calculations, total ADP and ALOS were recorded. Admissions per Year = ADP*365/ALOS. Death Rate per Person Year = (Deaths/ADP) × 100. Death Rate per 100,000 Admissions = (Deaths × 100,000)/Admissions per Year. This table includes three deaths occurring in FY2018 before ICE began routinely posting death reports to their website. FY2018–2020 data was previously published in AIMS Public Health [4].

A summary of characteristics from FY2018–2023 of individuals who died of suicide and primary medical causes are included in Table 2. The demographic characteristics of individuals who died did not differ significantly between study periods among the overall population (Supplementary Appendix 1) or among medical deaths specifically (Table 3). Suicide was identified as the cause of death in 1 of 8 (12.5%) cases in FY2021–2023 compared with 9 of 38 (23.7%) cases in the prior study period. Comparison characteristics of deaths by suicide between the two study periods are included in Supplementary Appendix 2.

Among primary medical deaths, COVID-19 was identified as contributory in 3 of 11 (27.3%) cases in FY2021–2023, compared to 8 of 11 (72.7%) cases occurring after April in FY2020 (p = 0.030) (Table 3). Cardiac arrest due to a primary medical condition for individuals in the detention facility was the cause of death in 4 of 11 (36.3 %) primary medical deaths during the recent study period, compared to 6 of 29 (20.7%) during the prior study period. Death reports indicate that EMS was notified, and CPR was initiated in 4 of 4 (100%) cases in the recent study period, and in 2 of these 4 (50%) cases there is indication that an AED was applied to the individual in the detention facility. Circumstances surrounding one of the in-facility cardiac arrest deaths are described in the illustrative case study, Exhibit 1.

Exhibit 1. **Illustrative Case of in-Custody Death in ICE Detention Facility.**

In December 2020, a 51-year-old man in ICE custody died at a detention facility in Mississippi. The man had a history of hypertension and hyperlipidemia, and, at the time of death, he had been in custody for approximately 14 months. On the day of his death, he was evaluated by facility staff for a complaint of chest pain and received an electrocardiogram (EKG), oxygen therapy, aspirin, and nitroglycerin. Documentation reveals that a physician reviewed the EKG and advised the nurse to monitor the individual for an hour before sending him back to his facility housing unit. One hour later, the individual was found unresponsive. EMS was activated, and facility medical staff initiated CPR measures until EMS arrival and subsequent pronouncement of death. Following the patient's autopsy, the cause of death was determined to be due to atherosclerotic cardiovascular disease.

This case was described in two reports issued by the Office of the Inspector General (OIG): (1) an investigation of deaths occurring in ICE and Customs and Border Patrol (CBP) custody during FY2021 [17], and (2) a report describing violations of ICE detention standards at the facility in which the individual died [18]. Reports reveal that the EKG obtained and reviewed by the facility physician demonstrated significant changes from an EKG performed one year prior. According to the OIG report, “based on a review of medical records and the autopsy report, our medical contractor concluded that...had medical staff compared the 2019 EKG with the one conducted on December 17, 2020, it should have prompted the medical staff to call 911 and send the detainee to the hospital, where life support care would have been readily available” [17]. Additionally, the physician contracted by OIG to review this case found that “care for this episode was not appropriate” and further determined that “the delay in getting the detainee to a higher level of care potentially contributed to his death” [17].

**Table 2.** Characteristics of individuals that died in ICE detention overall, FY2018–2023.

Characteristic	Total Study Period FY2018–2023			
	Total Deaths	Suicide	Medical	P value^e^
Subjects, No. (%)	50	10 (20)	40 (80)	
Age, Mean (SD) [range], y	47.0 (13.0)	40.1 (16.1)	48.8 (11.7)	0.058
	[21.3–75.0]	[21.3–75.0]	[22.6–73.0]	
Gender				
Male	46 (92)	10 (100)	36 (90)	0.571
Female^a^	4 (8)	0 (0)	4 (10)	
ICE detention, median (IQR) [range], d^b^	36.5 (10–86)	81.0 (10–119)	32.5 (9–81.5)	0.069
	[1–522]	[1–343]	[1–522]	
Location of Death, No. (%)				
Prehospital setting	10 (20.0)	6 (60)	4 (10)	0.002
Emergency department^c^	8 (16)	1 (10)	7 (17.5)	1
Hospital inpatient setting	32 (64)	3 (30)	29 (72.5)	0.024
Hospital days, median (IQR) [range], d^d^ (n = 32 hospitalized)	8.0 (4.5–21.5)	4.0 (1.0–8.0)	9.0 (5.0–23.0)	0.026
	[1–140]	[1–8]	[1–140]	

Note: ^a^Includes one individual whose sex was listed in the death report as male but who identified as female; ^b^Duration in ICE custody refers to the number of days in ICE custody prior to death or terminal hospital transfer; ^c^Deaths pronounced within one hour of arrival to hospital emergency department; ^d^Includes number of calendar days an individual was hospitalized following their terminal hospital transfer from detention facility, conditional on hospitalization; ^e^Student's t-tests were used to compare ages, negative binomial regression was used to compare length of time in ICE custody and duration of hospitalization among those hospitalized at death. Fischer's exact tests were used to compare proportions.

**Table 3.** Characteristics of individuals that died in ICE detention of primary medical causes by study period.

Characteristic	By Category of Cause of Death			
	Total Deaths	FY2021–23	FY2018–20	P value^e^
Subjects, No. (%)	40	11 (27.5)	29 (72.5)	
Age, Mean (SD) [range], y	48.8 (11.7)	46.9 (9.0)	49.5 (12.6)	0.544
	[22.6–73.0]	[36.0–61.0]	[22.6–73.0]	
Gender				
Male	36 (90)	10 (90.9)	26 (89.7)	1.000
Female^a^	4 (10)	1 (9.1)	3 (10.3)	
Cardiac arrest	10 (20)	4 (36.3)	6 (20.7)	0.418
COVID-19	11 (22)	3 (27.3)	8 (27.6)^f^	1.000
ICE detention, median (IQR) [range], d^b^	32.5 (9.0–81.5) [1–522]	37.0 (4.0–181) [1–442]	29.0 (14.0–78.0) [1–522]	0.211
Location of Death, No. (%)				
Prehospital setting	4 (10)	3 (27.2)	1 (3.4)	0.056
Emergency department^c^	7 (17.5)	2 (18.2)	5 (17.2)	1.000
Hospital inpatient setting	29 (72.5)	6 (54.5)	23 (79.3)	0.137
Hospital days, median (IQR) [range], d^d^ (n = 29 hospitalized)	9 (5.0–23.0) [1–140]	8.5 (5.0–20) [1–66], n = 6	11.0 (5.0–27.0) [1–140], n = 23	0.858

Note: ^a^Includes one individual whose sex was listed in the death report as male but who identified as female; ^b^Duration in ICE custody refers to the number of days in ICE custody prior to death or terminal hospital transfer; ^c^Deaths pronounced within one hour of arrival to hospital emergency department; ^d^Includes number of calendar days an individual was hospitalized following their terminal hospital transfer from detention facility, conditional on hospitalization; ^e^Student's t-tests were used to compare ages, negative binomial regression was used to compare length of time in ICE custody and duration of hospitalization among those hospitalized at death. Fischer's exact tests were used for proportions; ^f^COVID-19 was not recognized until mid FY 2020, and was responsible for 8 of 11 (72.7%) medical deaths occurring after April 2020, the difference between FY2021–2023 and COVID-era FY2020 deaths was p = 0.030 using Fischer's exact test.

In addition to the deaths described above reported by ICE in published death reports, the study team identified four reports of individuals who died shortly after release from ICE detention during FY2018–FY2023 [9],[12],[16],[19]. The median age of these individuals was 34.5 (range 25–55 years), and three identified as male and one as transgender female. Three were released from custody while hospitalized and deemed to be in a critically ill condition. In one instance, a hospitalized individual with a poor prognosis and in a protracted critical state was formally released from ICE custody without notification to the individual's family nor legal representative regarding the patient's status, hospitalization, or release. Notification of the patient's subsequent death three days following release from custody was also not provided to the patient's family or legal representative until a missing person's report was filed. Additional details surrounding this individual's death are included in Exhibit 2.

Exhibit 2. **Illustrative Case Study of Death after Release from ICE Custody.**

In March 2021, a 55-year-old male died three days after formal release from ICE custody during a prolonged hospitalization 12 months after entering ICE custody. During his initial intake screening, a review of his medical history was notable for diabetes mellitus, hypertension, hepatitis C, schizophrenia, and wheelchair dependence [11],[16]. He had been deemed incompetent to represent himself during deportation proceedings due to his symptoms of schizophrenia [16]. The individual was a class member in a lawsuit filed by the American Civil Liberties Union of Southern California (ACLU SoCal), *Roman v. Wolf*, which demanded a reduction in the ICE detention center population to meet federal social distancing guidelines during the COVID-19 pandemic [20],[21]. A federal court ordered ICE to reduce the population to a constitutionally permissible level in October 2020 [22], but, despite requests on his behalf, the individual in question was not among those released [21]. The individual ultimately contracted COVID-19 in December 2020 while detained in custody, and died in March 2021 after a series of hospitalizations related to complications of COVID-19 [23].

In February 2021, the month prior to his death, the detention facility's medical director notified the ICE Field Medical Coordinator that the individual was “at great risk of pulmonary embolism and [that there was a] possibility of sudden death due to multiple ailments, including ongoing weakness and chest pain in the wake of COVID-19 infection” [23]. A week later, he suffered a devastating stroke and was intubated, sedated, and reliant on ventilator support [23]. While hospitalized in critical condition, ICE formally released the individual from custody [11],[23]. Five days after his stroke and three days after his release from ICE custody, the man died alone in the hospital [16] from complications of COVID-19 [23].

Subsequent details of the case reveal that ICE notified this individual's attorney that they were considering his release from custody [11] on February 22^nd^ without a clear time frame. ICE formally released this individual over a week later on March 5^th^. Three days later on March 8^th^ he died without notification to his attorney or children [11],[16],[23]. The man's lawyer only learned of release from custody after receiving a message from a colleague who had seen the client's name on a list of individuals released from ICE custody [11],[23]. Concerned about the client's whereabouts, the man's lawyer contacted homeless shelters, police stations, and the coroner's office [23]. Ultimately, the lawyer learned of her client's death only after filing a missing person's report seven days after his death [11],[23].

Following the individual's death, the judge from his ACLU SoCal class action lawsuit ordered a Special Master to investigate ICE's conduct surrounding his death. The Special Master issued a report with several determinations and recommendations [23]. Principally, the Special Master concluded that the only practical effect of the individual's “release” from detention while he was comatose and near death was that he was moved “off the books” from the responsibility of ICE, the detention facility, and the organization contracted to provide medical services at the detention facility, and effectively moved onto the hospital's “books.” The report notes that, on release from ICE custody to the hospital, the detention center entities were relieved of their obligations to report subsequent outcomes, including death. The Special Master concluded that the sole purpose of the individual's release from ICE custody was effectively an avoidance of these obligations. Furthermore, the report concluded that an ICE representative should have followed longstanding policies and practices of notifying the individual's attorney of the plan for the man to be released to the hospital in anticipation of impending death [23]. Following the review of the Special Master's report and recommendations, the judge ordered the government (1) to report any instance when a detained individual at the detention facility who tested positive for COVID-19 dies, is confined to the infirmary, or is transported to the hospital for any reason; (2) to review the medical records of any detained individual who has previously tested positive for COVID-19 and is subsequently hospitalized or confined to the infirmary to determine whether they are being treated for COVID-19 or related complications, and if so to report that information; and (3) as a sanction for aforementioned conduct, to pay the man's attorney's legal fees for the period between when he was released, and when the attorney discovered his death [23]. The family of this man has since filed a lawsuit in response to his death [24]. Additionally, in 2021, ICE issued a directive establishing notification, review, and reporting requirements for deaths of individuals in detention [3]. Specifically, the directive reaffirmed policies to notify relevant parties and report the death of any detained person in a timely manner, and to ensure notification of the next of kin, appropriate consulate, relevant DHS and ICE stakeholders, Congress, and the public [3].

## 4. Discussion

The number of deaths and overall death rate among individuals in ICE detention decreased in FY2021–2023 compared to the prior COVID-era peak in FY2020. The number and proportion of deaths related to COVID-19 has also decreased since FY2020. Since the prior study period, COVID-19 vaccines have become available which drastically reduce adverse outcomes including hospitalizations and deaths [25]. Given that COVID-19 deaths have persisted for persons in ICE custody in the post-vaccine era suggests that individuals in ICE detention may benefit from guaranteed access to preventive measures, such as vaccination of all detained persons in confinement.

Notably, the lowest death count and rate was identified in FY2022, the year with the lowest reported average daily population (ADP) and average length of stay (ALOS). Additionally, the highest death count and death rate was identified in FY2020 when the ALOS was approximately 2–3 weeks longer than any other year during the study period. A lower ADP in detention facilities may allow for improved staff to detained person ratios and facilitate more reliable and expedient access to medical care, particularly in light of reported issues with vacancies in medical staffing of detention facilities [26]. Furthermore, a lower ADP may facilitate compliance with social distancing recommendations, thus reducing exposure to COVID-19 and other potentially fatal transmissible diseases.

The decrease in death rate from FY2021–2023 could also be related to recent federal court rulings that ordered ICE to release individuals from detention to protect them from contraction of COVID-19 due to the increased infectious disease transmission of confined spaces. In 2020, *Fraihat v. ICE* mandated the release of individuals with certain chronic health conditions from detention if they could not be “adequately protected’ from COVID-19. *Roman v. Wolf* resulted in the release of hundreds of people from the Adelanto ICE Processing Center in Southern California. *Zepeda Rivas v. Jennings* led to the release of individuals in ICE facilities in Northern California, among others. While many medically vulnerable individuals across the country were initially released under the *Fraihat v. ICE* ruling before it was overturned by the US Court of Appeals for the Ninth Circuit, the requirements of this ruling were not consistently implemented throughout detention facilities nationwide [27]. For example, in a Civil Rights and Civil Liberties complaint filed in August 2021, several medically high-risk individuals who were disproportionately Black, were denied release at Stewart Detention Center in Georgia [27]. Additionally, the individual described in Exhibit 2 was kept in ICE custody where he contracted COVID-19 and ultimately died from related complications. Notably, numerous medical comorbidities categorized this individual at high risk, and, although he was a class member in the *Roman v. Wolf* litigation, ICE denied his request for release. Had this individual been released prior to in-custody contraction of COVID-19, there may have been a different outcome.

It is notable that the individual identified in Exhibit 2 was formally “released” from ICE custody to a hospital after he was intubated, sedated, and ventilator-dependent shortly before his death. The individual suffered from several documented serious medical conditions and had been previously hospitalized for complications related to COVID-19. ICE did not notify his family nor his legal representatives, who only learned of his release through an acquaintance and confirmed his death after filing a missing person's report. A Special Master investigation of the man's death as part of a federal lawsuit raised questions as to whether the release of this hospitalized individual who carried a grave prognosis was influenced by an incentive by ICE to avoid reporting an additional in-custody death as mandated by the DHS [23]. Advocates and journalists have noted this pattern of deathbed releases, and argue that ICE may be motivated by the desire to avoid the financial and reputational harm associated with an in-custody death [10],[11],[16]. When individuals with terminal conditions may warrant compassionate release, prompt notification of such prognoses should be made to family members and legal representatives. In October 2021, ICE issued a directive regarding “Notification, Review, and Reporting Requirements for Detainee Deaths” [3]. The directive addresses notification of next of kin, appropriate consulate, Congress, and the public about all deaths of individuals in ICE detention *or* post release “when a death occurs within a reasonable time, not to exceed 30 days of release from ICE custody and review is requested by the ICE director” [3]. However, since the Congressional mandate in FY2018, ICE has not included death reports of individuals that died shortly after release from ICE custody on the *“Detainee Death Reporting”* website [2]. The language addressing an explicit requirement of notifications regarding post-release deaths (e.g., “it is ICE policy to the extent possible, to notify relevant parties and report the death of any detainee timely, accurately, appropriately, and with sufficient detail”) in the aforementioned directive could be interpreted in a number of ways, including private communications with the affected parties vs. public communications via ICE death reports. Furthermore, the directive is nonspecific in detailing a mechanism of accountability, stating, “ICE will conduct the required medical, oversight, and compliance reviews and investigations following the death of a detainee in ICE custody” [3]. While ICE has remained accountable to DHS requirements to publicly report all in-custody deaths, prompt notification regarding decompensation of health while in ICE custody and plans for pending release of the detained individuals is critical information for families and legal representatives. All out-of-custody deaths occurring within 30 days of release could be included in future mandatory public reporting of statistics and death reports by ICE. Further clarification of language in future directive could ensure reporting of post-release deaths.

In FY 2021–2023, 1 in 3 (33%) deaths resulted from cardiac arrest of a primary medical cause within a detention facility, an increase from 1 in 7 (14%) deaths in FY2018–2020. Death reports indicate that EMS was notified, and CPR was initiated in each of the 4 most recent cases, however only 2 death reports describe application of AEDs in facilities. As described in Exhibit 1, one individual died after experiencing cardiac arrest in a detention facility one hour after a physician reviewed an EKG to evaluate the complaint of active chest pain. Investigations by the OIG determined that the EKG should have prompted medical staff to request EMS for immediate transfer to a hospital, and that the delay in transporting the individual to a higher level of care potentially contributed to his death [17],[18].

ICE's Current National Detention Standards (NDS) details standards of training for management of “first aid and medical emergencies” to include recognition of potential emergencies, administration of CPR, recognition of mental illness, and plan for emergency care inclusive of transfer [28]. However, the NDS does not detail which levels of staff require such training, the frequency of training, standardized usage of AEDs, or mandatory maintenance of certifications in such fields as Advanced Cardiovascular Life Support (ACLS) [28]. Findings of this study suggest that, beyond compliance with the NDS, facilities must ensure that their staff has adequate training in management of basic life support measures (e.g., CPR), equipment (e.g., AEDs), and proficiency in protocols to escalate and secure transport to a higher level of care for individuals requiring advanced resuscitation. This is supported by recently published work evaluating EMS transports from ICE detention facilities, which suggests that transparency in emergency care provision in this setting is a critical component of ensuring appropriate delivery of care [29]. Future iterations of NDS might further strengthen the care of individuals experiencing medical emergencies at detention facilities by requiring on-site nurses including nurse practitioners, physician assistants, and physicians to maintain certification in ACLS, and to demonstrate appropriate skills and qualifications to recognize (e.g., EKG interpretation) and respond to (e.g., activate EMS) urgent and emergent situations that indicate the need for hospital transfer.

Several limitations must be recognized. First, this study is limited to individuals who died while in ICE custody and does not include information on individuals who died while in the custody of United States Customs and Border Protection (CBP) [20]. Second, death reports have not been posted for individuals released by ICE just prior to death as official “*Detainee Death Reports*” [2],[10]. A cross-reference of reports from independent advocacy organizations and media outlets throughout FY2018-2023 identified several instances where individuals hospitalized while in ICE custody and given a poor prognosis were subsequently released from custody and died shortly thereafter. Therefore, the calculated death rate underestimates deaths related to immigrant detention. Third, this exploratory evaluation of available information on ICE detention deaths is not adequately powered to detect differences in characteristics between groups, though study periods and group characteristics were compared as part of the descriptive exploration. Furthermore, detailed information about the demographics of individuals admitted into ICE custody during study years is not publicly available at this time, and therefore it is not possible to ascertain how shifting demographics might have influenced trends in mortality. However, this study represents an analysis of the best available data on deaths in ICE detention between FY2018 and FY2023.

## 5. Conclusions

Death rates in ICE detention have decreased from the pandemic-era peak of FY2020, along with a notable decline in COVID-19-related deaths. However, the persistence of COVID-19-related deaths in the post-vaccine era suggests that individuals detained by ICE may benefit from improvements in access to preventive measures, such as vaccination. The decrease in death rate overall may be attributable in part to federal court rulings in 2020 (e.g., *Fraihat v. ICE*, *Roman v. Wolf, Zepeda Rivas v. Jennings*) stipulating the release of medically vulnerable individuals from ICE custody and mandating consideration of specific vulnerabilities of individuals in determining whether to continue detention during the pandemic. Still, findings highlight instances of medically vulnerable individuals who died of complications of COVID-19 and were not granted release. It is notable that the protections granted in the *Fraihat v. ICE* decision have since been overturned [30]. Emerging trends in this study period are consistent with an increased proportion of deaths due to cardiac arrest in detention facilities, which highlights the importance of training requirements to ensure standardized resuscitation efforts. Additionally, the identification and release of medically complex individuals from ICE custody just prior to death indicates that calculated death rates underestimate total deaths associated with ICE detention. Ongoing monitoring of outcomes of individuals released from ICE custody is warranted.

## Use of AI tools declaration

The authors declare they have not used Artificial Intelligence (AI) tools in the creation of this article.


