# Supplementary material for: Deaths in Immigration and Customs Enforcement (ICE) detention: A Fiscal Year (FY) 2021–2023 update
Source: AIMS Public Health. 2024 Feb 27;11(1):223–35. doi: 10.3934/publichealth.2024011 (PMC11007418; doi:10.3934/publichealth.2024011)
Supplement: Supplementary file 1 [file publichealth-11-01-011-s001.pdf]

Public Health

AIMS Public Health, 11 (1): 223–235.
DOI: 10.3934/publichealth.2024011
Received: 06 December 2023
Revised: 01 February 2024
Accepted: 06 February 2024
Published: 27 February 2024

http://www.aimspress.com/journal/aimsph

Research article

Deaths in Immigration and Customs Enforcement (ICE) detention: A Fiscal Year (FY) 2021–2023 update

Cara Buchanan[1], Sameer Ahmed[2], Joseph Nwadiuko[3,4], Annette M. Dekker[5], Amy Zeidan[6], Eva Bitrán[7], Thomas Urich[2], Briah Fischer[8], Elizabeth R.E. Burner[2], Parveen Parmar[2] and Sophie Terp[2,*]

1 Department of Emergency Medicine, Harvard Medical School, Harvard University, Boston, MA, USA
2 Department of Emergency Medicine, Keck School of Medicine, University of Southern California, Los Angeles, CA, USA
3 Department of Medicine, UCLA David Geffen School of Medicine, Los Angeles, CA, USA
4 Department of Health Policy and Management, UCLA Fielding School of Public Health, Los Angeles, CA, USA
5 Department of Emergency Medicine, UCLA David Geffen School of Medicine, Los Angeles, CA, USA
6 Department of Emergency Medicine, Emory University School of Medicine, Atlanta, GA, USA
7 American Civil Liberties Union of Southern California, Los Angeles, CA, USA
8 Department of Obstetrics and Gynecology, University of Southern California, Los Angeles General Medical Center, Los Angeles, CA, USA

* **Correspondence:** Email: terp@usc.edu.

Supplementary

Appendix 1. Characteristics of individuals that died in ICE detention overall, by study period.

Characteristic	Total Study Period FY2018–2023			
	Total Deaths	FY2021–2023	FY2018–2020	P value[e]
Subjects, No. (%)	50	12 (24)	38 (76)	
Age, Mean (SD) [range], y	47.0 (13.0) [21.3–75.0]	44.9 (11.0) [23.0–61.0]	47.7 (13.6) [21.3–75.0]	0.524
Gender				
Male	46 (92)	11 (91.7)	35 (92.1)	0.571
Female[a]	4 (8)	1 (8.3)	3 (7.9)	
Suicide	10 (20)	1 (8.3)	9 (23.7)	0.416
Medical	40 (80)	11 (91.7)	29 (76.3)	
Cardiac arrest	10 (20)	4 (33.3)	6 (15.8)	0.225
COVID-19	11 (22)	3 (25)	8 (21.0)[f]	1.000
ICE detention, median (IQR) [range], d[b]	36.5 (10–86) [1–522]	111.00 (6-145) [1–442]	68.8 (10–86) [1–522]	0.272
Location of Death, No. (%)				
Prehospital setting	10 (20.0)	3 (25)	7 (18.4)	0.686
Emergency department[c]	8 (16)	2 (16.7)	6 (15.8)	1.000
Hospital inpatient setting	32 (64)	7 (58.3)	25 (65.8)	0.735
Hospital days, median (IQR) [range], d[d] (n = 32 hospitalized)	8.0 (4.5–21.5) [1–140]	8.0 (5.0–20.0) [1–66]	8.0 (4.0–23.0) [1–140]	0.833

Note: [a]Includes one individual whose sex was listed in the death report as male but who identified as female; [b]Duration in ICE custody refers to the number of days in ICE custody prior to death or terminal hospital transfer; [c]Deaths pronounced within one hour of arrival to hospital emergency department; [d]Includes number of calendar days an individual was hospitalized following their terminal hospital transfer from detention facility, conditional on hospitalization; [e]Student's t-tests were used to compare ages, negative binomial regression was used to compare length of time in ICE custody and duration of hospitalization among those hospitalized at death. Fischer's exact tests were used to compare proportions; [f]COVID-19 was not recognized until mid FY 2020, and was responsible for 8 of 12 (66.6%) overall deaths occurring after April 2020, the difference between FY2021–2023 and COVID-era FY2020 deaths was $p = 0.039$ using Fischer's exact test.

Appendix 2. Characteristics of individuals that died in ICE detention by suicide, by study period.

Characteristic	Update Years FY2021–2023			
	Total Deaths	FY2021–2023	FY2018–2020	P value[e]
Subjects, No. (%)	10	1 (10)	9 (90)	
Age, Mean (SD) [range], y	40.1 (16.1) [21.3–75.0]	23.0	42.0 (15.8) [21.2–75.0]	NA
Gender				
Male	10 (100)	1 (100)	9 (100)	NA
Female[a]	0 (0)	0 (0)	0 (0)	
ICE detention, median (IQR) [range], d[b]	81.0 (10–119) [1–343]	109	76.0 (10–119) [1–343]	0.889
Location of Death, No. (%)				
Prehospital setting	6 (60)	0	6 (66.7)	0.40
Emergency department[c]	1 (10)	0	1 (18)	1.00
Hospital inpatient setting	3 (30)	1 (100)	2 (11.1)	0.30
Hospital days, median (IQR) [range], d[d] (n = 7 hospitalized)	4.0 (1.0–8.0) [1–8]	8	2.5 (1–4) [0–4]	0.041

Note: [a]Includes one individual whose sex was listed in the death report as male but who identified as female; [b]Duration in ICE custody refers to the number of days in ICE custody prior to death or terminal hospital transfer; [c]Deaths pronounced within one hour of arrival to hospital emergency department; [d]Includes number of calendar days an individual was hospitalized following their terminal hospital transfer from detention facility, conditional on hospitalization; [e]Student's t-tests were used to compare ages, negative binomial regression was used to compare length of time in ICE custody and duration of hospitalization among those hospitalized at death. Fischer's exact tests were used to compare proportions.

AIMS Press

© 2024 the Author(s), licensee AIMS Press. This is an open access article distributed under the terms of the Creative Commons Attribution License (http://creativecommons.org/licenses/by/4.0)
